# Survey on currently applied interventions in neonatal resuscitation (SCIN): A study protocol

**DOI:** 10.3389/fped.2022.1056256

**Published:** 2023-01-09

**Authors:** Falk Eckart, Maxi Kaufmann, Colm P. F. O’Donnell, Lars Mense, Mario Rüdiger

**Affiliations:** ^1^ Saxony Center for Feto/Neonatal Health, Medical Faculty, TU Dresden, Dresden, Germany; ^2^ Neonatology & Pediatric Intensive Care Medicine, Department of Pediatrics, Medical Faculty, TU Dresden, Dresden, Germany; ^3^ Department of Neonatology, National Maternity Hospital, Dublin, Ireland; ^4^ School of Medicine, University College Dublin, Dublin, Ireland

**Keywords:** neonatal resuscitation, delivery room management, post-natal transition, supportive interventions, study protocol, neonatology

## Abstract

**Introduction:**

Around 140 million children are born every year and post-natal transition is uncomplicated in the vast majority. However, around 5%–15% of neonates receive supportive interventions during transition. Recent data on the interventions used is scarce. More data on the frequencies with which these interventions are used is needed to evaluate neonatal resuscitation, guide recommendations and to generate hypotheses for further research. The following protocol describes an international, multicentre survey on the interventions currently applied during neonatal resuscitation.

**Objectives:**

To determine the frequencies at which different supportive interventions recommended by European Resuscitation Council (ERC) guidelines for neonatal resuscitation are used. To compare the frequencies between hospitals and patient groups and to investigate possible factors influencing any differences found.

**Methods:**

Participating hospitals will collect data on all interventions performed during neonatal resuscitation over a period of 6 months. All hospitals providing perinatal care are eligible regardless of size and designated level of neonatal care. Every neonate requiring more interventions than basic drying and tactile stimulation during the first 30 min of life will be included. The targeted sample size is at least 4,000 neonates who receive interventions. After anonymization, the data is pooled in a common database and descriptive and statistical analysis is performed globally and in subgroups. Possible correlations will be investigated with phi coefficient and chi square testing.

**Ethics and dissemination:**

Consent of the institutional review board of the Technical University Dresden was obtained for the local data collection under the number BO-EK-198042022. Additionally, approval of local ethical or institutional review boards will be obtained by the participating hospitals if required. Results will be published in peer-reviewed journals and presented at suitable scientific conferences.

## Introduction

The transition to extra-uterine life is the most complex adaptation in life, involving major cardiorespiratory, hemodynamic and metabolic changes (1, 2). The majority of infants undergo this transition without any problems. However, it is estimated that up to 16% of newborns receive supportive interventions in the delivery room (DR) (3–5). With around 140 million births per year worldwide, this makes neonatal resuscitation and support of transition one of the most frequently performed medical interventions (6, 7).

The published frequency of medical interventions in the DR is based on limited data which are more than 20 years old (3, 8, 9). More recent data were mostly obtained from single centres in a certain region and do not take into account important risk factors like gestational age or mode of birth (10–14). Therefore, more current data on the frequency of DR-interventions are urgently needed, reflecting not only different national health systems and levels of care but also accounting for certain risk factors.

We aim to describe current practice in postnatal support by prospectively surveying clinicians at many hospitals internationally. Information will be collected on each DR-intervention that is performed at each participating centre for a period of 6 months. The data will be analysed regarding possible influencing factors both patient specific (e.g., gestational age, mode of birth) and structural (e.g., level of neonatal care, centre size).

### Objectives

The overall aim of this survey is to estimate the frequency of various interventions applied to newborn infants in the DR and to investigate the impact of possible influencing factors such as centre size and location, level of neonatal care, working hours and weekly periods.

The main objectives of this study are:

1. To estimate the total frequency of interventions given to neonates in the DR in order to support neonatal transition and to compare this estimate with prior data.
2. To estimate the frequency of single supportive interventions applied to neonates in the DR.
3. To investigate the impact of the following patient-specific factors on supportive interventions:
  a. gestational age (separated into different gestational age groups)
  b. mode of birth (c-section vs. vaginal delivery)
4. To investigate the impact of the following structural factors on frequency of single interventions:
  a. Designated level of neonatal care according to the definition of the American Academy of Pediatrics (AAP) (15)
  b. Centre size (defined by total number of newborn infants in 2021)
  c. Presence of trained neonatologists 24/7
  d. Centre location (defined by country)
5. To investigate the impact of the following organisational factors on frequency of supportive interventions:
  a. Weekly period of birth (Monday–Friday vs. weekend)
  b. Daily period of birth (00.00–08.00, 08.00–16.00, 16.00–00.00)

Further objectives include:

6. To further elucidate the mode of respiratory support by analysing the combined frequency of the use of CPAP, non-invasive ventilation and invasive ventilation, and by analysing the frequency of primary intubation (without application of non-invasive respiratory support).
7. To generate current data regarding surfactant administration in the DR by comparing the frequency of less-invasive surfactant administration versus application *via* endotracheal tube.
8. To investigate the frequency of laryngeal mask use as an alternative airway management tool in the DR.
9. To further elucidate the current practice regarding the route of drug administration by comparing the frequency of peripheral venous, umbilical venous and intra-osseous access in the DR.
10. To evaluate certain indicators of adherence to ERC guidelines (e.g., oxygen supplementation in resuscitation of early pre-term infants).

## Methods and analysis

### Study design

The study is an international, prospective and observational multi-centre survey. Anonymous data on interventions administered in the DR will be acquired once per patient. Follow-up data collection on the included patients is not planned.

### Study population/inclusion and exclusion criteria

Supportive DR-interventions are surveyed for all neonates who fulfil the following criteria:

◾ born in a participating hospital during the 6-month survey period
◾ receive supportive interventions more than basic drying or tactile stimulation during the first 30 min of life

Neonates are excluded based on the following criteria:

◾ neonates receiving only suction or peripheral venous access without any other supportive intervention
◾ neonates requiring supportive interventions only after the first 30 min of life.

### Sample size

The targeted sample size was decided upon empirically. The rarest described intervention in the literature is the administration of epinephrine with a frequency of as low as 0.05% and we wanted to collect data on at least 20 infants receiving this intervention. Therefore, the calculated total number of births during the study period must equal or exceed 40,000. We expect at least 500 births per participating hospital during the study period, leading to a target of 80 participating hospitals. Assuming an intervention rate of 10%, this results in a sample size of 4,000 infants receiving interventions. As we wish to be able to strive to have meaningful comparisons between different levels of neonatal care and different healthcare systems, we are looking to include hospitals from 20 countries and if possible multiple hospitals of different neonatal care levels per country. Ideally, this would mean 4 hospitals per country, one per level of neonatal care. Participating hospitals will be recruited *via* direct contact, referrals by other hospitals and presentation of the study protocol at scientific congresses.

### Survey sites

The survey has been developed and is coordinated by the Saxony Center for Feto/neonatal Health (SCFNH) at the Medical Faculty of TU Dresden. All hospitals where care is provided to newborns according to one of the levels of neonatal care defined by the American Academy of Pediatrics or a similar regional equivalent are eligible to participate, regardless of location and size.

The enrolment of hospitals is possible from 1st of March 2022 onwards. After successful enrolment, the participating hospitals will collect data over a period of 6 months. A follow-up to the study is not planned. Participating sites will be considered to have completed the survey-period when they have provided the data collected over 6 months to the common database.

### Study variables

For every included neonate the following data are collected:

• Weekly period of birth (Mo–Fr/Weekend)
• Time period of birth (8:00–16:00/16:00–0:00/0:00–8:00)
• Completed week of gestation
• Mode of birth (c-section/no c-section)
• Application of the following items during neonatal resuscitation:
  ○ Respiratory support:
    ◾ Initial inflations
    ◾ CPAP
    ◾ Non-invasive ventilation
    ◾ Oxygen supplementation
  ○ Airway management:
    ◾ Suction*
    ◾ Laryngeal mask
    ◾ Endotracheal intubation + invasive ventilation
  ○ Drug application:
    ◾ Surfactant administration (*via* endotracheal tube)
    ◾ Less-invasive Surfactant administration (LISA)
    ◾ adrenaline
    ◾ Caffeine
  ○ Vascular access
    ◾ Peripheral IV access*
    ◾ Umbilical venous access
    ◾ Intraosseous access
  ○ Chest compressions (if performed at least once)

Marked items (*) will only be recorded if performed together with other interventions due to practicability aspects. Tactile stimulation is not recorded because while it is most likely applied to every neonate, the management varies widely between centres and there exists no consensus on the most effective stimulation method (16).

For detailed definitions of the variables see Table 1.

**Table 1:** Definitions of study variables/data collection form items.

Study variable	Definition
Initial inflations	Positive pressure application using a facemask (at least ≥10 cm H_2_O over PEEP) over at least 2 s at least once before any other respiratory support
CPAP	Continuous positive pressure application (≥5 cm H_2_O) with sufficient device positioning and regardless of interface and pressure driver
Non-invasive ventilation	Intermittent positive pressure ventilation (iPPV) at least two times in a row regardless of interface and pressure driver; initial inflations excluded
Suction	Use of any suction device for airway clearance
Laryngeal mask	Insertion of laryngeal mask as airway device
Oxygen supplementation	Application of any additional oxygen, FiO_2_ > 0.21
Intubation + invasive ventilation	Ventilation *via* endotracheal tube (ETT); not to be ticked if intubation was used as mean for surfactant administration and the infant was extubated prior to the end of neonatal resuscitation
Surfactant *via* ETT	Surfactant administration *via* endotracheal tube, regardless of continuation of invasive ventilation after Surfactant administration
Less-invasive surfactant administration	Any other form of Surfactant administration less invasive than *via* endotracheal tube
Peripheral i.v. access	Placement of peripheral IV access regardless of administration of fluids/drugs
Umbilical venous access	Placement of umbilical venous access regardless of administration of fluids/drugs
Intraosseous access	Placement of intraosseous access regardless of administration of fluids/drugs
Chest compressions	Administration of chest compressions at least for one cycle (three compressions) according to ERC guidelines
Adrenaline	Administration of adrenaline at least once, regardless of administration route

Additionally, the following baseline characteristics are collected for every survey site:

• Designated level of neonatal care according to the definition of the AAP (15)
• Total births per year (historical data from 2021)
• Number of admissions to the neonatal intensive care unit (either in-house or nearest available) per year (historical data from 2021)
• Total number of infants born per month during the survey period
• Total number of infants born per month *via* c-section during the survey period
• Permanent availability of a trained neonatologist either in-house or on call

### Data collection and management

All survey sites will collect the observational data prospectively into the provided Data Collection Form (see Attachments), either in paper or electronic form. Data missing due to documentation errors can be obtained by retrospective chart review. The completed Data Collection Form will be sent electronically on a monthly basis to the SCFNH for entry into the central database (CD). If assigned, local patient IDs will be removed before transferral to the SCFNH in order to provide full anonymization in the CD. All survey sites will receive an ID for center pseudonymization before being included in the CD. The CD will be stored password-protected locally within the SCFNH and can only be accessed by members of the study team. As the data is collected in an anonymized way, no separate consent form is obtained. Possibly missing/unclear data will be requested from the participating hospitals. In case the requested data cannot be provided, the respective data will be marked and not included in statistical analysis.

### Data analysis

Data will be analysed globally and for specific, predefined subgroups for every independent variable (e.g., completed week of gestational age, mode of birth, designated level of neonatal care).

Descriptive analysis will be performed on all study variables including absolute numbers and percentages as well as median and range for numerical variables. For all observed interventions confidence intervals will be calculated on a confidence level of 0.95. Additionally, statistical analysis regarding possible correlations will be performed on all study variables using phi coefficient and chi square test of independence. Statistical analyses and graphical presentations will be performed using R (The R Foundation for Statistical Computing, Vienna, Austria).

## Anticipated results

We are expecting that the targeted sample size of at least 4,000 neonates receiving interventions will be collected within 1 year of enrolling the first hospital. To reduce bias, we are looking to collect data on equal amounts of births for every level of neonatal care and to include a hospital of every level of neonatal care from every participating country.

The minimum of anticipated results of descriptive and statistical analysis is listed in Table 2. Further results of additional analysis might be added if the need emerges during the data analysis process. Selected results of descriptive analysis and significant statistical analysis will be presented in graphical form as diagrams. All results regardless of significance will be presented in tabular form, at the least as supplementary material of the planned publication.

**Table 2:** List of anticipated results of descriptive and statistical analysis.

Descriptive analysis	Statistical analysis
Total intervention frequency (global and stratified into subgroups sorted by mode of birth, level of neonatal care, center size, country, weekly period of birth, daily period of birth)	Significant differences in total intervention frequency between the stratified subgroups (mode of birth, level of neonatal care, center size, country, weekly period of birth, daily period of birth)
Frequency of single supportive interventions (global and stratified into subgroups sorted by gestational age, mode of birth, level of neonatal care, center size, country, weekly period of birth, daily period of birth)	Significant differences in single supportive intervention frequency between the stratified subgroups (mode of birth, level of neonatal care, center size, country, weekly period of birth, daily period of birth)
Combined frequency of CPAP, non-invasive ventilation and invasive ventilation and frequency of primary intubation (without application of non-invasive respiratory support)	Significant differences in single supportive intervention frequency for neonates receiving interventions stratified by gestational age groups

## Discussion

Supporting postnatal transition of neonates seems to be the most frequently performed medical intervention (3). National and international guidelines are regularly updated in order to recommend an evidence based approach towards neonatal life support (17). However, data on the frequency of different interventions is scarce, outdated and often localized and therefore not representative for different health care systems or levels of care.

This international and multi-centre survey will provide much needed data on the frequency with which interventions are currently applied in the DR. Furthermore, the survey will examine the influence of different factors on administered interventions. Thereby, it will not only provide up-to-date information for future guidelines but will also help to generate hypotheses for further research and designing future studies on DR-management.

Current guidelines recommend different interventions in order to support postnatal transition, with a special focus on respiratory support (17). As a result, non-invasive positive pressure ventilation is a relatively common intervention with described frequencies ranging from 4% to 12% of all neonates (14, 18). Other interventions like the administration of chest compressions or epinephrine are much rarer at described frequencies from 0.1% up to 0.3% (3, 18) and 0.05% to 0.3% (14, 19) respectively. However, little is known for other interventions.

Video recordings of neonatal resuscitation represent a possible solution to track performed interventions (20). By detailed analysis of video recordings, certain DR-interventions in preterm and term infants after c-section were previously described both for a single centre and as a comparison between different centres (21–23). However, this data is not representative for all infants.

The multi-centre and international approach of surveying current DR-management allows a comparison across centres of different sizes, levels of neonatal care and health care systems. The survey is easy to perform and not very time consuming, thus it is easy for hospitals to participate, ensuring large data sets. The prospectively planned stratification into different subgroups will provide detailed information for certain risk groups. Comparison of data from different countries will disclose variations that could be due differences in local guidelines, recommendations and practices.

Whereas the survey has its strengths, its design has some potential limitations. Due to the observational design results should be interpreted with care and no causal relationships can be analysed. As no detailed information regarding the application order of the performed interventions is collected, a possible causal influence between different interventions is not detectable. Conducting the survey in a Data Collection Form approach enhances practicability but prevents data collection on the intensity of interventions (e.g., pressure levels, oxygen concentrations, duration of application). Furthermore, the medical indication for performed interventions is not recorded as well as other potential confounders such as maternal characteristics. Lastly, due to the multi-centre nature and the strict anonymization, quality control measures against under-reporting can only be undertaken on a local level.

## Ethics and dissemination

The survey will be conducted according to the International Ethical Guidelines for Health related Research involving humans issued by the Council for International Organizations of Medical Sciences (24), the ethical principles in the Declaration of Helsinki (25) and any applicable local laws and regulations. Formal consent of the institutional review board of the Technical University Dresden was obtained under the number BO-EK-198042022. Additionally, approval of local ethical or institutional review boards is obtained by the participating hospitals if required. Furthermore, the survey is registered in the German Register for Clinical Studies (Deutsches Register Klinische Studien, DRKS) under reference DRKS00029736.

For dissemination purposes the results of the survey will be published in peer-reviewed journals and presented at suitable scientific conferences. It is also planned to give individual feedback to the participating hospitals and to benchmark their data to the global dataset.

## Data Availability

The original contributions presented in the study are included in the article/Supplementary Material, further inquiries can be directed to the corresponding author.
